# Effect of inappropriate admissions on hospitalization performance in county hospitals: a cross-sectional study in rural China

**DOI:** 10.1186/s12962-019-0176-5

**Published:** 2019-04-02

**Authors:** Jing-jing Chang, Ying-chun Chen, Hong-xia Gao, Yan Zhang, Hao-miao Li, Dai Su, Di Jiang, Shi-han Lei, Xiao-mei Hu, Min Tan, Zhi-fang Chen

**Affiliations:** 1 School of Medicine and Health Management, Tongji Medical College, Huazhong University of Science and Technology, Wuhan, 430030 Hubei China; 2 Research Center for Rural Health Services, Hubei Province Key Research Institute of Humanities and Social Sciences, Wuhan, 430030 Hubei China

**Keywords:** Excessive utilization of health services, Inappropriate admissions, Propensity score matching, Appropriateness evaluation protocol

## Abstract

**Background:**

Inappropriate admissions cause excessive utilization of health services compared with outpatient services. However, it is still unclear whether inappropriate admissions cause excessive use of health services compared with appropriate admissions. This study aims to clarify the differences in the hospitalization performances between appropriately admitted inpatients and inappropriately admitted inpatients.

**Methods:**

A total of 2575 medical records were obtained after cluster sampling in three counties. Admission appropriateness was assessed by appropriateness evaluation protocol (AEP). The propensity score matching (PSM) was computed to match patients in treatment and control group with similar characteristics, and to examine the differences in the utilization of hospitalization services between the two groups. The samples were matched in two major steps in this study. In the first step, total samples were matched to examine the differences in the utilization of hospital services between the two groups using 15 individual covariates. In the second step, PSM was computed to analyze the differences between the two groups in different disease systems using 14 individual covariates.

**Results:**

For the whole sample, the inappropriate group has lower expenditure of hospitalization (EOH) (difference = − 0.12, p = 0.003) and shorter length of stay (LOS) (difference = − 0.73, p = 0.016) than the appropriate group. For number of clinical inspection (NCI), it has no statistically significant difference (difference = − 0.39, p = 0.082) between the two groups. Among different disease systems, no significant differences were observed between the two groups among EOH, LOS and NCI, except that the EOH was lower in the inappropriate group than that in the appropriate group for surgical disease (difference = − 0.169, p = 0.043).

**Conclusion:**

Inappropriate admissions have generated excessive health service utilization compared with appropriate admissions, especially for internal diseases. The departments in charge of medical services and hospital managers should pay high attention to the health service utilization of the inappropriately admitted inpatients. Relevant medical policies should be designed or optimized to increase the appropriateness in health care service delivery and precision in clinical pathway management.

## Background

Excessive use of health services leads to waste of health resources and unreasonable increase in medical costs. It is an issue of widespread concern across the globe. Inappropriate admission is one of the most severe problems in the excessive use of health services [1]. Inappropriate admission refers to a condition that the utilization of hospitalization services is not conducted on the basis of clinical needs [2], and physicians act as patients’ agents and therefore can influence the choice of patients to use hospitalization services compared to outpatient services. Therefore, patients tend to choose the hospitalization services according to the physicians’ advice. Admission appropriateness can be assessed by appropriateness evaluation protocol (AEP), which is an objective, effective and reliable tool used to evaluate the appropriateness of the admissions on the basis of inpatient’s medical records [3–6]. AEP criteria can be divided into two parts: medical service intensity and disease severity.

Some studies have pointed out that compared with outpatient services, inappropriate admissions cause excessive use of health services, including human resources, beds, medicines and health care funds [7]. However, it is not clear whether inappropriate admissions cause excessive use of health services compared with appropriate admissions. Though previous studies have indicated that patients with less severe diseases tend to have shorter length of stay (LOS) in hospital, and therefore, consume less health resources [8, 9], this does not mean that the relatively small amount of health resources are consumed by inappropriately admitted patients. The reasons are as follows.

First of all, patients’ actual utilization of hospitalization services will be affected by policies that related to inpatient service delivery in the hospital such as standardized inpatient service provision policy and clinical pathway management and so on. No matter admission is appropriate or not, the patient will receive health care services in accordance with the standards of hospitalization service process. In other words, medical treatments, nursing and examination are strictly implemented in accordance with the clinical pathway form [10], where the timing of diagnosis and treatment measures is clarified, the clinical process is programmed, and the inspection, treatment and nursing that should be done every day are clearly specified. The patient would receive corresponding clinical biochemistry inspection, for instance blood test, urine test [11], and they would spend a standard period in hospital, and receive the prescribed dosage. In this way, it may result in a condition that inappropriately admitted patients have the similar utilization of hospital services compared to those who are admitted appropriately. Second, the patient’s condition is in constant change with uncertainty. The change of the patient’s condition will affect the subsequent series of health services [12]. For instance, the symptoms of the appropriately admitted inpatients may relieve rapidly after hospitalization and require less treatment methods, and accordingly, the length of stay may be shortened. Patients who are admitted inappropriately may get worse, when the utilization of medical services will be more intense. In this case, the length of stay may be extended and the expenditure of hospitalization will increase. Thirdly, the utilization of health services is greatly influenced by doctor’s behaviors. On the one hand, according to the prospect theory proposed by Kahneman [13], most people are risk-averse when facing gain and risk preference when facing loss. People are more sensitive to loss than to gain. As a result, people are often wary of taking risks in the face of gains. In other words, irrespective of the appropriateness of admission, doctors may tend to adopt the most conservative treatment methods when they are not sure of the situation in the process of diagnosis and treatment. This leads patients to do more clinical inspections, extend length of stay for observation and so on, all of which will increase the use of health services. On the other hand, doctor’s behaviors will also be affected by the hospital internal management system and salary system. This generates behaviors that induces consumption or reduces service, etc. Thus, the relationship between the appropriateness of admission and the utilization of health services is unclear, which calls for further exploration.

A study of Bianco A has revealed that doctors adopt a conservative management mode due to their risk aversion, which led to inappropriate admission and inappropriate follow-up hospitalization services [14]. This increased the length of stay, resulting in unnecessary waste of resource. It is more common in surgery departments. Velasco’s [15] study illustrated that patients who were not properly admitted had three times the length of hospital stay compared to those admitted appropriately. Eriksen [16] has measured the proportion of inappropriate admission of internal medicine and the cost, and the findings suggested that not accepting inappropriate admission did not bring the hospital the same percentage of cost reduction.

Although some studies have explored the relationship between admission appropriateness and hospitalization services utilization, these studies also have some limitations. First, some of studies were based on the comparison of an inpatient case itself. These studies were the evaluation of appropriateness of admission and services utilization after hospitalization. They were not compared with other similar or opposite cases. Second, although length of stay and hospitalization expenses were studied in the evaluation of health services utilization, there were little detailed studies on the utilization of clinical inspection. Third, and most importantly, insufficient consideration of the severity of disease made the comparison of service utilization lack of accuracy. The difference in disease severity and the change of disease condition will affect the utilization, and therefore, the performance of hospitalization services. Inappropriate admission and those with appropriate admission both have patients with mild and serious severity. Whether there is a difference in the utilization of services, and if so, what are the differences in the efficiency of hospitalization service are still unknown, and await further study. In addition, matching patients with similar disease characteristics is of great practical significance for accurate evaluation of medical quality, utilization of health services and rational allocation of health resources.

Based on AEP, the appropriateness of admission is evaluated from two aspects of medical service intensity and disease severity. The admissions rated as “inappropriate” indicate that the patients have a mild illness, and the required medical service intensity for them is not large. Thus, in theory, the consumption of health resources for inpatients admitted inappropriately is smaller than those admitted appropriately. Thus, this study hypothesized that inappropriate inpatients have shorter length of stay (LOS), fewer number of clinical inspection (NCI), and lower expenditure of hospitalization (EOH) than those who are admitted appropriately and with similar characteristics, and to verify it. The main contribution of this study relative to other similar studies is the adoption of propensity score matching (PSM) methodology. The PSM was used to match patients in appropriate and inappropriate admissions with similar characteristics, and to examine the differences in the utilization of hospitalization services among them. This would enrich more contribution relative to other methods.

This study is expected to provide reference for the policy makers when adjusting and improving relevant medical policies in order to promote the appropriateness of health resources utilization and control unreasonable increase in hospitalization expenses.

## Methods

### Data source

Three counties were selected as the sample counties (Dingyuan in Anhui province in central China; Huining in Gansu province, Yilong in Sichuan province in western China). The reimbursement and payment levels of the new rural cooperative medical scheme (NRCMS) in the three counties are similar.

Cluster sampling method was applied in this study. The largest and most capable public comprehensive hospital in each county was selected as a sample hospital. Medical records were the objects of sampling. In the sampling calculation, according to the existing research [2], the estimated inappropriate admission rate P is 16%, and the relative tolerance δ = 0.09, the absolute tolerance *d* = 0.09 * *P* = 1.44%, the significance level α = 0.05, and the one-sided standard normal deviation Z_α_ = 1.96. The equation of sample size (N) was as follows:

1 $${\text{N}} = ({{\text{Z}}_{\upalpha}}/{\text{d}})^{2} \times {\text{P}} (1 - {\text{P}}) = (1.96/1.44\%)^{2} \times 16\% \times (1-16\%)=2489.93$$

**Fig. 1:**
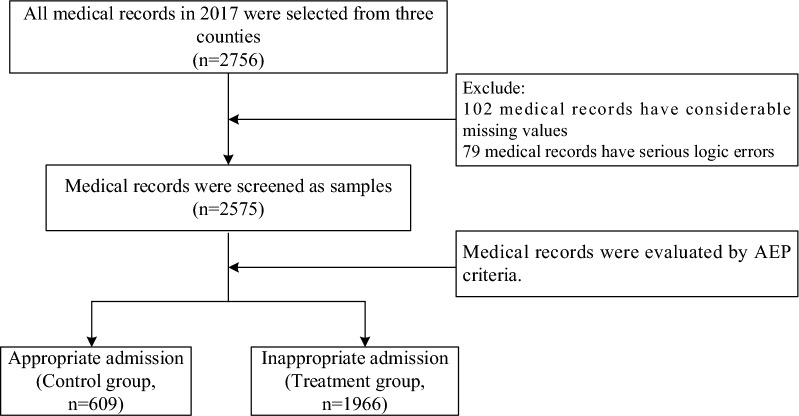
Study design and flow chart of the medical records selection and the classify of those medical records in two groups

Considering the quality of medical records, 900 medical records in 2017 were selected from each hospital. Firstly, admissions of hospital delivery records in obstetrics were excluded considering the pertinence of AEP. Then, corresponding quantity of medical records were selected from the remaining departments according to the proportion of patients in the department accounted for the total quantity of patients in all departments. At last, a total of 2575 medical records were screened as samples after eliminating the records that have too many missing values and serious logic errors (Fig. 1). There were no missing values in outcome variables in the final samples.

All the medical records were evaluated by an adjusted AEP standard constructed in 2014 for county hospitals in China [17] (Appendix). The records were evaluated by two trained judges respectively. The judges were members of the research team. A professional training was held before they evaluating the admission appropriateness. Among all the records, 609 admissions were regarded appropriate (the control group) and 1966 were classified as inappropriate (the treatment group). This study believes that in addition to the general influencing factors (individual basic characteristics, external systems and policies, etc.) that affect the utilization of inpatient services, the constant development and change of the disease itself is also an important factor that cannot be ignored. Based on the above considerations, this paper used a dynamic perspective to compare the utilization of health services after hospitalization in patients that with different admission appropriateness. This is also one of the highlights of this study. Based on such a research perspective and the characteristics of the AEP criteria, the medical records were judged mainly according to the patients’ indications at the time of admission (when some disease indications may not be fully manifested) rather than the final discharge results (when the disease indications are relatively comprehensive). Because disease indications are not fully manifested, it may be not easy to meet AEP’s criteria for “appropriate” admission. On this basis, it is possibly lead to overestimating the inappropriate admission rate.

### Study variables

#### Outcome variables

In this study, we use LOS, NCI and EOH as the outcome variables. These three indicators can be used to describe the patients’ utilization of services. LOS is a comprehensive index that directly measures hospital medical quality and management level [18]. NCI is an important index to reflect services projects of inpatients receiving. EOH is a critical index in the evaluation of health economics, which is the most direct reflection of health resource consumption [19]. At the same time, considering that EOH may not conform to the normal distribution, the study logarithmically processed variable EOH and it conformed to the normal distribution after logarithmic transformation.

#### Explanatory variables

Since the selection of covariates by PSM was to include relevant variables that may affect the outcome variables and processing variables as far as possible to satisfy the negligible hypothesis, this study included as many covariates as possible in the medical records. There were 15 patient-level covariates in the study, including gender, age, type of medical insurance, profession, marital status, way of admission, frequency of hospitalization, department in charge of treatment, disease system, having more than one disease, status of the patient upon admission, history of disease, with chronic diseases, health condition at ordinary times and receiving any surgery. Due to disease severity and considerations different, differences exist in the utilization of health services among different age. Type of medical insurance also affects the utilization of health services. Especially with the development of NRCMS, the reimbursement ratio increases gradually, which promotes the release of patient medical service demand and increases the services projects [2]. The profession may affect the length of hospital stay. For instance, farmers may shorten the LOS regardless of the severity of the disease during busy seasons [20, 21]. Health condition at ordinary times, status of the patient upon admission, having more than one disease and disease system are closely related to the changes of patients’ conditions after hospitalization. These are variables that especially need to be paid attention to in this study. Changes in illness can affect LOS and utilization of services [22]. Whether receiving any surgery would influence their hospitalization results due to the risk of nosocomial infections and complications [23].

### Propensity score matching (PSM)

There were differences in individual characteristics between the treatment and control group, which will affect the comparison of the results of service utilization. Propensity scores were used to match each inpatient between two groups in similar conditions. PSM was used to balance observable covariates and reduce potential selection bias [24, 25]. The samples were matched in two major steps in this study. In the first step, total samples were matched to examine the differences in the utilization of hospital services between two groups using 15 individual covariates. In the second step, PSM was computed to analyze the differences in different disease systems, because the use of health services varies among disease systems. Disease system was divided into five groups (circulatory diseases, digestive diseases, respiratory diseases, surgical diseases and others). Then, inpatients in the treatment and control group were matched in each group of disease. Fourteen individual covariates were used except “disease system”. Therefore, it can be known whether there are significant differences in service utilization between the two groups in different diseases systems.

### Statistical analysis

First of all, propensity score was obtained by incorporating the covariates into the logit model. Then, kernel matching was used to match each patient in the treatment group with similar counterpart patients in the control group (one-to-one matching) based on propensity score. The matching result of kernel method is good in terms of accuracy and it was summarized through literature in the field of health services [24, 26]. Finally, we calculated the average treatment effect on treated (ATT), which reflects the average change level of the outcome variable after controlling the covariates.

Assume that each inpatient *i* has two potential outcomes, $Y_{i1}$ (treat, inappropriate admission) and $Y_{i0}$ (control, appropriate admission). The average effect of the treatment is given by $E(Y_{i1} - Y_{i0} )$. However, as $Y_{i0}$ and $Y_{i1}$ cannot be observed simultaneously for the same inpatient, the ATT is calculated instead:

2 $$ATT = E(Y_{i1} |D_{i} = 1) - E(Y_{i0} |D_{i} = 1)$$

where $D_{i}$ is the dichotomous indicator of treatment, with 1 indicating that inpatients *i* are admitted inappropriately, and 0 are admitted appropriately. Stata 15.0 software (Stata Corp LP, College Station, TX, USA) was used for statistical analysis in a Windows environment. The two-sided statistical significance level was set at 0.05.

## Results

### Basic characteristics of the sample

As shown in Table 1, the three outcome variables were significantly different between the two groups (p < 0.01), and the mean value and standard deviation of treatment group was lower than that of the control group. Covariates were also significantly different (p < 0.05), except for gender, marital status, and frequency of hospitalization variables.

**Table 1:** Distribution of characteristics of admission cases

Variables	Treatment group	Control group	Sig
	(N = 1966)	(N = 609)	
Outcome variables			
Expenditure of hospitalization (EOH)	7.85 ± 0.89	8.06 ± 0.970	< 0.01
Length of stay (LOS)	7.22 ± 5.74	8.22 ± 7.062	< 0.01
Number of clinical inspection (NCI)	7.98 ± 4.68	8.56 ± 4.825	< 0.01
Explanatory variables			
Gender			> 0.05
Male	1029 (52.34)	332 (54.52)	
Female	937 (47.66)	277 (45.48)	
Age	45 ± 26.46	49 ± 23.96	< 0.01
Type of medical insurance			< 0.05
Medical insurance for urban workers	397 (20.19)	105 (17.24)	
Medical insurance for urban residents	58 (2.95)	16 (2.63)	
NRCMS	1123 (57.12)	330 (54.19)	
Medical assistance	281 (14.29)	117 (19.21)	
Others	107 (5.44)	41 (6.73)	
Profession			< 0.01
Peasantry	902 (45.88)	271 (44.50)	
Student	529 (26.91)	207 (33.99)	
Others	535 (27.21)	131 (21.51)	
Marital status			> 0.05
Spinsterhood	455 (23.14)	120 (19.70)	
Married	1475 (75.03)	482 (79.15)	
Others	36 (1.83)	7 (1.15)	
Department in charge of treatment			< 0.01
Pediatrics	400 (20.35)	69 (11.33)	
Internal medicine	674 (34.28)	215 (35.30)	
Surgery	390 (19.84)	111 (18.23)	
Others	502 (25.53)	214 (35.14)	
Frequency of hospitalization	1 ± 1.08	1 ± 0.83	> 0.05
Disease systems			< 0.01
Circulatory diseases	308 (15.67)	110 (18.06)	
Digestive diseases	427 (21.72)	95 (15.60)	
Respiratory diseases	515 (26.20)	77 (12.64)	
Surgical diseases	434 (22.08)	231 (37.93)	
Others	282 (14.34)	96 (15.76)	
Having more than one disease			< 0.05
No	1841 (93.64)	584 (95.89)	
Yes	125 (6.36)	25 (4.11)	
Status of the patient upon admission			< 0.01
Dangerous	238 (12.11)	134 (22.00)	
Serious	196 (9.97)	55 (9.03)	
Urgent	1213 (61.70)	338 (55.50)	
General	319 (16.23)	82 (13.46)	
Receiving any conduct surgery			< 0.01
No	1669 (84.89)	482 (79.15)	
Yes	297 (15.11)	127 (20.85)	
Health condition at ordinary times			< 0.05
Fine	1340 (68.16)	385 (63.22)	
General	523 (26.60)	197 (32.35)	
Worse	103 (5.24)	27 (4.43)	
History of disease			< 0.01
No	1430 (72.74)	407 (66.83)	
Yes	536 (27.26)	202 (33.17)	
With chronic disease			< 0.01
No	1571 (80.11)	452 (74.22)	
Yes	395 (20.09)	157 (25.78)	
Way of admission			< 0.01
Outpatient	1382 (70.30)	379 (62.23)	
Emergency	584 (29.70)	230 (37.77)	

Data in the table: Mean ± standard deviation/Number (constituent ratio, %)

The test for continuous variables is independent samples t-test, and the test for categorical variables is Chi-squared test

### The matching effect and results of the PSM of the total samples

As Table 2 shows, the covariates of the treatment group and the control group were well balanced after matching (p = 0.928, mean bias = 1.7, median bias = 1.3).

**Table 2:** Overall balance test results of PSM

	Overall balance				
	Pseudo R^2^	LR chi^2^	p	Mean bias	Median bias
Raw sample before matching	0.032	88.97	0.000	10.6	11.7
Matched sample after kernel matching	0.001	7.90	0.928	1.7	1.3

In Table 3, in the whole sample, the EOH of treatment group was lower than that of control group (difference = − 0.12, p = 0.003) after matching. The control group has longer LOS than the treatment group (difference = − 0.73, p = 0.016). There was no statistically significant difference in the NCI between the two groups (difference = − 0.39, p = 0.082).

**Table 3:** Matching results of the PSM

Sample	Matching results			Bootstrap results			
	Difference	S.E.	T-stat	Difference	Z-value	p	95% CI (lower, upper)
Expenditure of hospitalization (EOH)							
Raw sample before matching	− 0.21	0.042	− 5.00				
Matched sample after kernel matching	− 0.12	0.047	− 2.51	− 0.12	− 2.92	0.003	− 0.20 to − 0.04
Length of stay (LOS)							
Raw sample before matching	− 1.00	0.282	− 3.55				
Matched sample after kernel matching	− 0.73	0.334	− 2.19	− 0.73	− 2.4	0.016	− 1.33 to − 0.13
Number of clinical inspection (NCI)							
Raw sample before matching	− 0.59	0.219	− 2.70				
Matched sample after kernel matching	− 0.39	0.236	− 1.65	− 0.39	− 1.74	0.082	− 0.83 to 0.05

All results are computed using the Stata module of psmmatch2

*S.E.* standard error, *CI* confidence interval

### The matching effect and results of the PSM of the samples in disease systems

As Table 4 shows, in disease systems, the covariates of the treatment group and the control group were well balanced after matching except respiratory disease (p = 0.093, mean bias = 4.60, median bias = 4.7).

**Table 4:** PSM matching effect and results of the disease systems

Sample	Overall balance					Matching results			Bootstrap results			
	Pseudo R^2^	LR chi^2^	P	Mean bias	Median bias	Difference	S.E.	T-stat	Difference	Z-value	p	95% CI (lower, upper)
Expenditure of hospitalization (EOH)												
Circulatory diseases	0.014	10.54	0.649	5.30	3.9	− 0.013	0.11	− 0.12	− 0.013	− 0.13	0.893	− 0.20 to 0.18
Digestive diseases	0.007	8.31	0.873	3.00	2.0	− 0.119	0.10	− 1.19	− 0.119	− 1.28	0.199	− 0.30 to 0.06
Respiratory diseases	0.015	21.35	0.093	4.60	4.7	0.031	0.13	0.24	0.031	0.24	0.812	− 0.23 to 0.29
Surgical diseases	0.003	3.46	0.998	2.70	2.0	− 0.169	0.10	− 1.77	− 0.169	− 2.02	0.043	− 0.33 to 0.01
Others	0.008	6.53	0.951	4.60	4.5	0.174	0.12	1.46	0.174	1.34	0.180	− 0.08 to 0.43
Length of stay (LOS)												
Circulatory diseases	0.014	10.54	0.649	5.30	3.9	− 0.198	0.78	− 0.25	− 0.198	− 0.23	0.820	− 1.90 to 1.51
Digestive diseases	0.007	8.31	0.873	3.00	2.0	− 0.607	0.68	− 0.89	− 0.607	− 0.87	0.384	− 1.97 to 0.76
Respiratory diseases	0.015	21.35	0.093	4.600	4.7	− 0.094	0.77	− 0.12	− 0.094	− 0.10	0.919	− 1.91 to 1.72
Surgical diseases	0.003	3.46	0.998	2.70	2.0	− 1.427	0.72	− 1.99	1.427	− 1.86	0.063	− 2.93 to 0.07
Others	0.039	30.24	0.007	7.70	5.5	− 0.592	1.07	− 0.55	− 0.377	− 0.29	0.773	− 2.94 to 2.18
Number of clinical inspection (NCI)												
Circulatory diseases	0.014	10.54	0.649	5.30	3.9	− 0.603	0.67	− 0.90	− 0.603	− 0.89	0.375	− 1.94 to 0.73
Digestive diseases	0.007	8.31	0.873	3.00	2.0	0.201	0.52	0.39	0.201	0.45	0.652	− 0.67 to 1.08
Respiratory diseases	0.015	21.35	0.093	4.600	4.7	− 0.089	0.63	− 0.14	− 0.089	− 0.17	0.868	− 1.14 to 0.89
Surgical diseases	0.003	3.46	0.998	2.70	2.0	0.129	0.45	0.29	0.129	0.33	0.741	− 0.64 to 0.90
Others	0.039	30.24	0.007	7.70	5.5	0.663	0.59	1.13	0.688	1.04	0.300	− 0.61 to 1.99

In EOH outcome variable, the treatment group was lower than that of control group in surgical disease (difference = − 0.169, p = 0.043). There was no significant difference in other disease groups (p > 0.05). In LOS and NCI outcome variables, there was no significant difference in all disease systems between two groups (p > 0.05).

## Discussion

Propensity score matching has been widely used in the field of health economics since the 1990s [27]. It eliminates the selective bias and the mixed bias by matching the individuals in the treatment group with the appropriate comparable objects in the control group [28]. In this study, the resource consumption between appropriate admission and inappropriate admission groups was compared by controlling the factors influencing the utilization of services. This method balances the problems caused by incomplete and inaccurate pairings [29]. Meanwhile, the results of multiple covariates acting together can be expressed [30]. This makes the results accurate and comparable.

For the whole sample, with the similar basic characteristics, the patients admitted inappropriately had shorter LOS and lower EOH than those admitted appropriately. As a whole, the study indicates that the health service utilization of patients admitted inappropriately was less than that of patients admitted appropriately. This is basically in line with expectations. No difference in NCI was observed between them. This indicates that inappropriate admission may result in overuse of clinical inspection services. In different disease groups, there were some specific differences. Diseases were classified into internal and surgical diseases. Among surgical diseases, the EOH of inappropriate admission group was less than the appropriate admission group. As long as the patient needs surgery, he/she is easy to accord with the AEP (A1, A4,) and be evaluated as an appropriate admission. The cost of surgery, drug exchange and infusion, etc., will make the EOH of the appropriate admission higher than that of the inappropriate admission. Thus, in terms of surgical diseases, inpatients admitted inappropriately will not consume more health resources than those admitted appropriately.

The circulatory diseases, digestive diseases and respiratory diseases are internal diseases. There was no statistically significant difference in internal disease in the EOH, LOS and NCI between the two groups. This is different from the comparison results of the surgical diseases. There are two possible explanations.

First, as organic disease, the cause of the internal diseases is complex and difficult to be identified and diagnosed [31]. It has hidden features, and the features of the disease may gradually become apparent after admission. In this case, the means of diagnosis and treatment may increase after hospitalization among the inappropriately admitted patients. Second, the symptoms of patients admitted inappropriately did not change after admission, but the consumption of resources was as much as that of patients admitted appropriately, which indirectly indicates that there exists avoidable consumption of health resources. It may be related to the salary system of public hospitals in China. For years, under the condition of market economy [32], doctors in public hospitals have been required to earn their own salary through business income. At the same time, the pricing of technical services is seriously low, and the human capital value of doctors is not fully reflected. The inappropriate salary structure and distribution factors has led to further distortion of incentive mechanism [33]. Most obviously, “subsidizing medical services with medicine” has distorted the behaviors of medical staff, making them prescribe “big prescriptions” to pursue the maximization of economic benefits to protect their vital interests. They prefer to use expensive drugs, let patients do more clinical inspections and extend the LOS of patients, resulting in increased hospitalization expenditures [34, 35]. Meanwhile, intense doctor–patient relationship at present has led to a condition that some medical staff conduct unnecessary clinical inspections and treatments on patients in order to avoid risks [36]. Besides, it is related to doctors’ own treatment habits and lack of grasp of disease severity [37]. It is possible that after the patient admitted to hospital, doctors provide the services that they are accustomed to, or treat patients according to the specified clinical pathways. Due to the lack of judgment on the severity of the disease, some services are unnecessary, especially for patients who are not appropriately admitted to the hospital.

To solve these problems, it is necessary to reform the salary distribution system of public hospitals [38]. Public hospitals should improve the incentive mechanism of internal allocation by making doctors earnings more on the value of their labor than the quantity of their services. The combination of effective motivation and supervision to the hospitals can promote the hospitals to improve efficiency, reduce service cost, shorten the LOS and reduce the induced expenditure. In addition, optimizing the clinical pathway management is necessary. The clinical pathway aims to optimize the service process, reduce the delay in disease treatment and waste of resources, and provide patients with efficient and high-quality medical and nursing services [39]. Even so, it does not indicate that there is no overconsumption of resources. Studies have shown that the effect of reducing hospital costs through clinical pathway management is limited [40, 41]. Clinical pathways can improve the treatment effectiveness, but it does not reduce the length of stay or hospital costs [42]. Therefore, when implementing clinical pathway, it is necessary to consider the same disease with different severity and make clinical pathway more elaborate.

### Limitations

This study has four limitations. At first, although PSM eliminates the selective bias and the mixed bias by matching the individuals, which makes the two groups more comparable, it only controls the influence of measurable variables, and “hidden bias” may still occur if selection on unobservable variables exists. Second, the content of covariate indicators is limited and cannot fully reflect the real situation of patients. Third, the medical records may not be accurate enough, which may affect the appropriateness evaluation. Finally, in the PSM process, kernel matching was selected to used. Although this method has good applicability in practice, there may be other more suitable matching methods.

## Conclusions

Inappropriate admissions have generated excessive health service utilization compared with appropriate admissions, especially for internal diseases. On the one hand, the NCI of inappropriately admitted inpatients had no significant difference compared with appropriately admitted inpatients on the whole. On the other hand, when it comes to the disease systems, no significant differences existed between the two groups among EOH, LOS and NCI, except that the EOH was lower among the inappropriate group than the appropriate group in surgical disease. Policy makers need to pay more attention to the utilization of health resources of inappropriately admitted inpatients. Relevant medical policies should be optimized to promote medical service providers’ appropriateness of health service provision, and the clinical pathway management should be more precise. At the same time, patients should be guided to utilize health services appropriately.

## Appendix

See Table 5.

**Table 5:** AEP criteria for county hospitals

A. Medical service intensity
A1. Need follow-up treatment within 24 h: (1) instruments or other facilities that are only available for hospitalised patients (angiography, visceral biopsy, cardiac catheterisation intervention) and/or (2) invasive diagnostic of central skeletal muscle meat (lumbar puncture, cisterna puncture, ventricular puncture, encephalography)
A2. Treatment with varying dosages or drugs on a regular basis under direct medical supervision
A3. Calculation of intake and output volume
A4. Operation to be conducted on the following day in the operating room, detailed pre-operative consultation or evaluation on the day of admission
A5. Main surgical incision and drainage nursing
A6. Quarantined patients
A7. Bedside electrocardiogram (ECG) monitoring or testing vital signs at least every 2 h
A8. Stopping (at least once every 8 h) or continuing oxygen inhalation
A9. Referral of post-operative recovery
B. Disease severity
B1. Continuous fever > 38.0 °C for more than 5 days
B2. Acute confusion (coma or adiaphoria)
B3. Severe anomaly in electrolyte or blood and vigour, showing the following situations: (1) Na < 123 mEq/L or > 156 mEq/L; (2) K < 2.5 mEqt/L or > 6.0 mEq/L; (3) HCO_3_ < 20 mEq/L or > 36 mEq/L; and (4) arterial blood pH < 7.30 or > 7.45
B4. Loss of sight or hearing for 48 h
B5. Loss of activity in any part of the body for 48 h
B6. Excretion disorder or absence of intestinal peristalsis in the past 24 h
B7. Active bleeding
B8. Needing blood transfusion because of bleeding
B9. Mental disorders caused by non-alcohol dependence
B10. Viscera removal or surgical wound dehiscence
B11. Pulse less than 50 or greater than 140 beats per minute
B12. Abnormal blood pressure: systolic blood pressure < 90 mmHg or > 200 mmHg and/or diastolic blood pressure < 60 mmHg or > 120 mmHg
B13. Ventricular fibrillation or acute myocardial ischemia shown by electrocardiogram (ECG) report or course log
B14. Acute hematopathy, severe medium-sized leukopenia, thrombocytopaenia, leukocytosis, erythrocytosis, thrombocytosis or haemolysis-resulted symptoms
B15. Progressive acute neurological disorders
B16. Soft tissue injuries affecting basic self-care
B17. Acute myocardial infarction or cerebrovascular accident (stroke)
B18. Spinal cord lesions
B19. Lung infection above 50% or leafy lesions according to X-ray examination
B20. Hyperemesis or acute pain caused by acute or chronic diseases

The AEP criteria for the county hospital was derived from the research results of a National Natural Science Foundation project undertaken by our research team. *(Research on Measurement and Management of Excessive Demand for Inpatient Service of the New Rural Cooperative Medical Scheme, NO. 71073061)*. It was based on the experience of international AEP criteria, and was combined with the reality of rural China. After several rounds of expert consultation and combined with field research, the AEP criteria for county hospital was accomplished and a monograph (Excessive demand for rural hospitalization service—a study on the measurement and management of inappropriate admission) has been published

## Abbreviations

AEP: appropriateness evaluation protocol
PSM: propensity score matching
EOH: expenditure of hospitalization
LOS: length of stay
NCI: number of clinical inspection
NRCMS: new rural cooperative medical scheme
